# Generative adversarial network-based creative synthesis and AI art creation in children’s art education: effects on health promotion

**DOI:** 10.3389/fpubh.2026.1945238

**Published:** 2026-10-07

**Authors:** Jing Chen, Yang Zhang, Hui Liu

**Affiliations:** College of the Arts Hebei Agricultural University, Baoding, China

**Keywords:** AI-assisted art education, artificial intelligence, children, creativity development, digital health, health promotion, human-AI collaboration, mental health

## Abstract

**Background:**

The existing research on children’s art education still has shortcomings in the field of artificial intelligence creation support and lacks an exclusive generation model and systematic evaluation framework that takes into account the characteristics of children’s cognitive development and physical and mental health needs, which limits the practical application of digital aesthetic education in children’s health promotion. Based on the development law of children’s aesthetic cognition and the needs of mental health promotion, this study constructs an artificial intelligence (AI) generation model for children’s artistic creation. By integrating children’s figurative thinking characteristics, creativity development laws and emotional expression needs, it realises accurate support for children’s artistic creation process. At the same time, a multi-dimensional evaluation system covering creativity, aesthetic literacy, mental health, visual health and social development is established to systematically evaluate the impact of AI-assisted aesthetic education on children’s comprehensive health development so as to provide a theoretical basis and practical reference for children’s health-orientated digital aesthetic education model.

**Methods:**

From July 2025 to December 2025, 120 children aged 6–14 (divided into three developmental levels: 6–8, 9–11, and 12–14) were selected for a 12-week controlled intervention experiment, and the experimental group (AI man–machine collaborative aesthetic education, using the optimised children-specific improved WGAN-GP creative synthesis model) was set up. In this study, a quasi-experimental design was adopted, and the intervention effect was inferred by the correlation between groups and the changes within groups, without strict causal attribution. In the statistical analysis, age is taken as a covariate into the ANCOVA model, and hierarchical subgroup analysis is carried out according to age levels to test the potential influence of age on the main results. Multidimensional statistical methods such as the independent sample *t*-test, paired sample *t*-test, two-factor repeated measurement variance analysis, one-way covariance analysis (ANCOVA) with age as a covariate, Pearson correlation analysis, intra-group correlation coefficient (ICC), standardised mean difference (SMD), and determination coefficient (r) were used. Through the Torrance Creative Thinking Test (TTCT), the self-made Children’s Artistic Aesthetic Literacy Rating Scale, which has been systematically developed and verified by reliability and validity, the generated image quality evaluation system, the Children’s Depression and Anxiety Scale (SCARED), the Children’s Perceived Stress Scale (CPSS), the visual fatigue score, the children’s social expression health scale, the naked eye vision difference and the resting heart rate, the multi-dimensional indicators, the observed image quality evaluation system was generated. All health scales are marked with the applicable age range, and the standardised testing procedures of picture-assisted and item-by-item reading are adopted for children aged 6–8, and the boundaries of adult assistance are defined.

**Results:**

Compared with the control group, the Fréchet dIStance (FID) of the experimental group is 21.15 ± 1.31, the inception score (IS) is 2.87 ± 0.16, and the anti-loss convergence rate is increased by 29.7%, so the model has excellent stability. The results of interpersonal intervention showed that the creativity fluency (79.68 ± 5.82), flexibility (77.35 ± 5.41), and uniqueness (75.46 ± 5.73) of the experimental group were significantly better than those of the control group (67.52 ± 5.13, 65.94 ± 4.87, and 63.15 ± 5.23). After controlling the age covariate, the difference between groups remained significant (*f* value ranged from 87.32 to 156.48, all *p* < 0.001), and the main effect of age was significant in some indexes but did not change the intervention direction. The scores of aesthetic perception, color matching, and composition creativity in the experimental group (84.36 ± 3.81) were significantly higher than those in the control group (66.28 ± 4.53), in which the scores of works with AI assistance and works without AI were reported separately to distinguish the quality of AI-assisted output from children’s independent aesthetic ability. The scores of anxiety (12.35 ± 2.11), perceived stress (9.62 ± 1.87), and visual fatigue (2.14 ± 0.63) in the experimental group were significantly lower than those in the control group (21.76 ± 3.42, 17.53 ± 2.69, and 4.89 ± 0.92). The fluctuation difference of naked-eye vision after class was 0.12/0.05 in the experimental group and 0.25/0.08 in the control group (*p* < 0.01). 5 mins after class, the resting heart rate was 77.93 ± 5.26 beats/min in the experimental group and 86.71 ± 6.22 beats/min in the control group (*p* < 0.01). The score of social health expression (87.41 3.65) was significantly higher than that of the control group (64.33 4.17), and the difference of all health indicators among groups was *p* < 0.001. The score consistency within the group was ICC = 0.84 (*p* < 0.001), and the intervention effect amount SMD = 0.68, which was a large-effect positive intervention. Pearson correlation analysis shows that children’s participation in AI creation is highly positively correlated with the quality of creative output (*r* = 0.812, *p* < 0.001), which is a cross-sectional correlation and does not constitute causal inference. Tool familiarity, personal preference, and exploration behavior may be potential confounding factors. Stratified subgroups showed that children with high anxiety and stress at baseline had the greatest improvement.

**Conclusion:**

This study shows that the optimized creative synthesis model of the generation confrontation network can adapt to children’s artistic creation thinking, and the improvement of creative thinking and aesthetic quality is observed in both AI-assisted creation and children’s independent creation, accompanied by the reduction of anxiety, psychological pressure, and visual fatigue and the improvement of social expression level. In view of the quasi-experimental design and limited sample size of this study, the above results should be understood as intervention correlation rather than strict causal effect, and the performance of works under AI-assisted conditions should not be directly equivalent to children’s independent aesthetic ability. This study provides a preliminary empirical basis for the scientific and standardized development of digital children’s health aesthetic education.

## 1 Introduction

In recent years, the rapid development of digital technology and artificial intelligence (AI) has been changing children’s education and art learning. AI generative painting, intelligent creative auxiliary systems and other technologies have gradually entered the school art education scene, providing new possibilities for promoting children’s creativity cultivation, aesthetic ability improvement and mental health support (1, 2). As an important representative of generative artificial intelligence, a Generative Adversarial Network (GAN) can realise autonomous composition, colour generation and creative expression by learning a large number of image data and provide diversified support for children’s artistic creation (3). However, at present, most AI art generation models are mainly trained based on adult art works and adult aesthetic preferences. Their generation logic is more in line with the aesthetic characteristics of mature cognitive groups and lacks targeted design for children’s cognitive development rules, creative expression methods and healthy development needs. Therefore, the applicability and health promotion value of the existing models in children’s art education still need to be further verified.

Childhood is a critical stage in the development of creativity, aesthetic ability, emotional regulation ability and social interaction ability (4). According to the theory of cognitive development, preschool and school-age children mainly think in concrete images and have distinct development characteristics in colour perception, spatial imagination and creative expression. Artistic creation is not only an important manifestation of children’s cognitive development but also an important way to promote emotional expression, relieve psychological pressure and enhance social adaptability. Studies have shown that positive artistic activities can improve children’s emotional state, improve their self-expression ability, and have a positive impact on mental health (5, 6). However, the existing GAN and its improved models mostly focus on image quality and generation accuracy as the main optimisation objectives and pay less attention to children’s artistic expression characteristics and their potential impact on psychological, visual and social health. In the context of children’s education, the lack of an exclusive generation model that takes into account the needs of creativity cultivation and health promotion limits the in-depth application of AI-assisted aesthetic education.

At the same time, the application of AI technology in children’s art education is still in the exploratory stage. The existing research focuses on the innovation of teaching modes or the effect of classroom practice and lacks systematic evaluation of the health effects of AI-assisted artistic creation (7, 8). Especially in children, the empirical evidence on whether AI-assisted aesthetic education can promote creativity development, improve emotional state, reduce psychological pressure and enhance social expression ability is still relatively limited. In addition, the current evaluation system mainly focuses on the teaching effect or model performance indicators and seldom integrates multidimensional health indicators such as creativity, aesthetic literacy, mental health, visual health and social function into a unified framework, which makes it difficult to comprehensively evaluate the comprehensive value of AI-assisted aesthetic education.

It should be pointed out that children aged 6–14 years cover the late pre-operation stage, the concrete operation stage, and the early formal operation stage, and there are significant developmental differences in artistic expression fineness, abstract thinking ability, and self-report reading comprehension. Therefore, age-level factors are included in the scale measurement, work grading standards, and statistical analysis in this study to control the potential impact of developmental heterogeneity on the results. The existing GAN-related research mainly focuses on image generation quality optimization, model stability improvement, industrial design, digital media, and other application fields (9, 10). In contrast, the research on the generative model for children is relatively insufficient, especially the lack of an interdisciplinary research framework integrating children’s developmental psychology, art pedagogy, and health promotion theory. Children’s cognitive characteristics, aesthetic preferences, and health needs have not been fully considered in the process of model design and effect evaluation, which makes it difficult for existing technologies to effectively serve children’s art education and healthy development practices.

This study is based on public primary schools and community aesthetic education centres in first-tier cities in South China, China. The subjects are mainly from families with medium or above socio-economic status, and their families have access to digital devices. Children have different degrees of experience in touching touch-screen devices and smart applications in their daily lives. This social and cultural background is of great significance to the interpretation of the research results: First, there has been a long-standing pattern of “aesthetic education marginalisation” and “exam-orientated” in the basic education system in China, and children’s schoolwork pressure is great, and their artistic creation time is compressed. The anxiety and pressure improvement observed in this study may partly reflect the compensation effect of artistic creation itself as an emotional outlet, rather than the specific role of AI technology; Secondly, families in first-tier cities have a higher acceptance of digital education products, and children’s initial familiarity with AI tools may be better than that of children of the same age in economically underdeveloped areas, which may affect the correlation strength between AI creative participation and creative output. Thirdly, children are highly sensitive to “collective creation” and “peer evaluation” in the cultural context of China. The improvement of the social expression health score in this study may be related to the group cooperation in the classroom and the cultural orientation of collectivism in China, rather than the social guidance function of AI-generated content itself. The above social, cultural, economic and educational background factors may affect children’s experience and evaluation of AI-generated works of art in three ways: first, family digital capital determines children’s pre-experience in contacting AI tools, which in turn affects the exploration depth and creative adoption rate in human-computer interaction; Secondly, the tradition of school aesthetic education and teachers’ teaching style have shaped children’s aesthetic expectations of “good works,” which may make them make a differentiated evaluation of non-realistic and highly saturated pictures generated by AI; Thirdly, different attitudes towards “technology-assisted learning” in regional cultures may affect children’s subjective perception of AI-assisted creation—whether AI is regarded as a “creative partner” or a “substitute tool”. Therefore, the transferability of the conclusion of this study should be carefully evaluated, and the moderating effect of the above background factors should be considered when it is promoted to children with different cultural and socio-economic backgrounds.

Based on the above research background, this study optimizes the WGAN-GP model based on the law of children’s cognitive development and health promotion needs and constructs a human–machine collaborative generation system suitable for children’s artistic creation. At the same time, a multi-dimensional evaluation system covering creativity, aesthetic literacy, mental health, visual health, and social expression ability was established, and the application effect of AI-assisted aesthetic education was evaluated by comparing the intervention and experimental systems. It should be noted that this study adopts a quasi-experimental design instead of a randomized controlled trial, and the sample size is relatively limited and comes from a specific social and cultural background. Therefore, the research results should be carefully interpreted within the scope allowed by the design and should not be excessively extrapolated to a wider group of children. The purpose of this study is to explore the potential value of children’s exclusive AI creative generation model in promoting artistic development and healthy growth and to provide empirical evidence for children’s health-oriented digital aesthetic education practice.

## 2 Methods

### 2.1 Research object and experimental design

This study selected 120 children from July 2025 to December 2025 to carry out a 12-week teaching intervention experiment, strictly followed the empirical research norms of pedagogy, and explored the intervention effect of an AI human–machine collaborative aesthetic education model supported by an improved WGAN-GP model on children’s artistic creative ability and aesthetic literacy. In this study, school-age children were selected as subjects, and the inclusion criteria and exclusion criteria were strictly set. The effective sample size was calculated by the sample size formula and then grouped to ensure that the experimental baseline was balanced and comparable between groups. Synchronously following the dual empirical research norms of pedagogy and children’s public health, the experimental group (AI human-machine collaborative aesthetic education) and the control group (traditional hand-painted aesthetic education) were set up, with 60 people in each group; all subjects completed standardized physiological and psychological screening before entering the group and collected two objective physiological data points of uncorrected visual acuity and resting heart rate every 4 weeks. Before and after the intervention, a complete set of international physical and mental scales was measured. According to the baseline SCARED and CPSS scale scores, the health stratification subgroup was divided, and the statistical analysis was carried out.

All children completed standardized physiological-psychological combined screening in a quiet environment in the school medical room one week before enrollment. The total duration of single screening was controlled within 10 min, and paper files were recorded synchronously by two people. Any screening item was judged to be positive and directly excluded, and it was not included in the sample. The psychological screening process was completed by a full-time psychological teacher, the guardian filled in the basic health questionnaire, and the child independently completed the simplified SCARED and CPSS pre-screening scales. The scale score reached the clinical critical value, or the interview found that the hidden emotions and social barriers were judged to be positive for psychological screening; physiological screening used a campus general standard logarithmic visual acuity chart and a household electronic bracelet to complete static naked-eye vision and a 5-min resting heart rate screening. At the same time, the fine copy function test of the hand was carried out, and the patients with corrected visual acuity <0.6, persistent arrhythmia, and hand movement disorder were directly excluded. All children with negative screening were allowed to enter the 12-week intervention experiment. The original screening data were archived separately, only for baseline balance matching, and did not participate in the post-intervention efficacy statistics.

#### 2.1.1 Age distribution and development level matching

In view of the extensive developmental span of 6–14 years old, which involves substantial differences in artistic expression, cognition, literacy, and self-reporting, this study divided the children into three developmental levels according to their age and ensured that the number of people in each level was balanced between the experimental group and the control group: the 6-8-year-old level (from the late pre-operation stage to the early concrete operation stage, *n* = 40, 20 in the experimental group/20 in the control group) and the 9-11-year-old level (22 people in the experimental group/22 people in the control group) and 12–14 years old (from the late stage of concrete operation to the early stage of formal operation, *n* = 36, 18 people in the experimental group/18 people in the control group). The chi-square test showed that there was no significant difference between the two groups in the distribution of people at all age levels (*χ^2^* = 0.187, df = 2, *p* = 0.911), which was comparable between the two groups. The average age, sex ratio, baseline TTCT score, and aesthetic literacy score of each age level are shown in Table 1. In the statistical analysis, age is taken as a covariant in the form of a continuous variable, and stratified subgroup analysis is carried out according to the above three levels to test the potential influence of age on the main results.

**Table 1:** Baseline characteristics of participating children stratified by age groups.

Age group	Indicator	Control group (*n* = 60)	Experimental group (*n* = 60)	Statistical parameter	*p* value
6–8 years	Number of participants (male/female)	20 (11/9)	20 (10/10)	*χ^2^* = 0.102	0.749
	Mean age (years)	7.15 ± 0.68	7.08 ± 0.72	*t* = 0.312, df = 38	0.757
	Baseline total TTCT score	189.4 ± 12.3	190.1 ± 11.8	*t* = 0.182, df = 38	0.856
	Baseline aesthetic score	64.8 ± 4.2	65.3 ± 4.0	*t* = 0.378, df = 38	0.708
9–11 years	Number of participants (male/female)	22 (12/10)	22 (11/11)	*χ^2^* = 0.092	0.762
	Mean age (years)	9.86 ± 0.82	9.91 ± 0.79	*t* = 0.198, df = 42	0.844
	Baseline total TTCT score	196.2 ± 13.1	195.8 ± 12.7	*t* = 0.102, df = 42	0.919
	Baseline aesthetic score	66.5 ± 4.5	66.8 ± 4.3	*t* = 0.224, df = 42	0.824
12–14 years	Number of participants (male/female)	18 (9/9)	18 (9/9)	*χ^2^* = 0.000	1.000
	Mean age (years)	12.94 ± 0.75	13.02 ± 0.71	*t* = 0.328, df = 34	0.745
	Baseline total TTCT score	201.5 ± 14.2	200.9 ± 13.8	*t* = 0.132, df = 34	0.896
	Baseline aesthetic score	67.2 ± 4.8	67.5 ± 4.6	*t* = 0.198, df = 34	0.844

Between-group comparisons within each age stratum were performed using independent-samples *t*-test for continuous variables and chi-square test for categorical variables. No statistically significant between-group differences were observed for all baseline indicators (*p* > 0.05), indicating balanced baseline comparability between the two groups across developmental age strata.

#### 2.1.2 Inclusion and exclusion criteria

*Inclusion criteria*: ① The age is 6–14 years old, which is in the stage of rapid development of children’s creativity and thinking in images; ② Good physical and psychological conditions, no nervous system diseases, obvious visual dysfunction, color vision defects, and other health problems that may affect artistic perception and creative performance; ③ No systematic professional art training and no long-term off-campus painting course or special art training experience so as to ensure that the subjects’ artistic foundation is relatively consistent; ④ Be able to participate in the whole 12-week intervention process as required; complete the teaching activities, assessment tasks, and works collection at all stages; and have an expected attendance rate not less than 90%; ⑤ Children’s guardians have fully understood the research purpose, implementation process, and data usage, voluntarily signed the informed consent form, and agreed to use the relevant data for scientific research and analysis.

*Exclusion criteria*: ① With attention deficit, hyperactivity disorder, cognitive developmental disorder, or other developmental problems that may affect classroom participation and artistic expression ability; ② There is serious abnormal color vision, impaired visual function, or hand motor dysfunction, so it is impossible to complete the painting creation task independently; ③ Participated in similar art education intervention projects or received special art-intensive training within 6 months before the start of the study; ④ It is estimated that it is impossible to maintain a high degree of participation, and there is a risk of long-term absence from class, so it is difficult to complete the prescribed intervention and data collection process; ⑤ The guardian disagrees to participate in the study, fails to cooperate with the follow-up evaluation, or voluntarily applies for withdrawal during the study.

#### 2.1.3 Sample size calculation

This study used a two-group parallel control teaching intervention design. The sample size was estimated according to the mean comparison formula of two independent samples. The two-sided significance level *α* = 0.05 and the test power (1 − *β*) = 0.80 were set, and the corresponding parameters were *Zα*/2 = 1.96 and *Zβ* = 0.84, respectively. According to the results of the preliminary experiment (a total of 30 children were included, 15 in the experimental group and 15 in the control group, and the samples were from the same school and the same grade), the standard deviation of children’s artistic creative literacy score was *σ* = 4.26, the expected minimum difference between the groups was *δ* = 3.15, and the calculated effect size d was approximately 0.74. The results showed that at least 40 subjects were included in each group to meet the statistical requirements. Taking into account the possible withdrawal of research and missing data during the intervention process, a 10% sample loss margin was added to the theoretical sample size. Finally, 60 children were included in each group, with a total sample size of 120. Admittedly, the sample size of 120 cases is relatively limited for the study covering the extensive developmental span of 6–14 years old, especially when stratified subgroup analysis is carried out according to age level and health status, the sample size of each subgroup is further reduced (the smallest subgroup *n* = 32), which may affect the statistical efficiency of subgroup analysis. Therefore, the stratified subgroup results of this study should be regarded as exploratory findings and need to be verified in a larger sample study. The sample size can meet the requirements of educational intervention research on statistical effectiveness and provide reliable data support for intergroup difference analysis and correlation tests. Finally, a total of 120 eligible children completed the study, including 60 in the experimental group and 60 in the control group. The results of baseline comparison showed that there was no significant difference in age, gender composition, artistic literacy level, or creative thinking ability between the two groups (*p* > 0.05), which had good inter-group comparability and laid a foundation for the analysis of follow-up intervention effect.

#### 2.1.4 Ethical review and informed consent

This study strictly abides by ethical norms and adheres to the research principles of voluntariness, harmlessness, confidentiality and fairness. The research scheme has been approved by the academic committee of our school. All the guardians of the children have been fully aware of the experimental period, intervention form, evaluation content, and data use scope of this study and voluntarily signed a written informed consent form. In order to fully protect the rights and interests of underage subjects, this study implemented a stratified informed consent procedure based on the cognitive understanding ability of children of different ages. For children aged 6–8 years old, the research team first used children’s easy-to-understand explanatory materials to introduce the research purpose, implementation content, and participation process in concise language. After obtaining the children’s oral or written willingness to participate (assent), their guardians signed the formal informed consent. For adolescents aged 12–14 years, the written informed consent of the subjects was obtained on the basis of the informed consent signed by the guardian. All subjects have the right of voluntary withdrawal and can unconditionally terminate their participation at any stage of the study without any adverse effects. During the research process, all data are anonymized and coded instead of personally identifiable information. The original data is only used for scientific research analysis, not disclosed to third parties, and not used for any commercial purposes, so as to maximize the protection of the privacy and security of the subjects and ensure that the research meets the requirements of ethical norms.

In this study, a parallel control teaching intervention design was adopted, and a 12-week intervention cycle was set up. The course was carried out twice a week, and each course lasted for 90 min. The two groups are consistent in terms of teacher allocation, teaching duration, course schedule, teaching theme, teaching environment, and teaching equipment, and there are only differences in core teaching intervention methods to ensure that only the impact of changes in teaching mode is examined during the research process. In order to reduce the risk of bias, the study used a single-blind design. The children did not understand the specific grouping. The evaluators and data analysts worked under blind conditions so as to effectively reduce the subjective evaluation bias and confounding factors and improve the objectivity and credibility of the research results.

#### 2.1.5 Baseline health stratification subgroup

With the median standardized scores of SCARED and CPSS as the stratified cut-off value, 120 children were divided into three subgroups. After the intervention, the objective physiological indexes and the improvement of the scale scores were compared: low anxiety and low pressure subgroup: the scores of the two scales were lower than the median, *n* = 42; medium physical and mental state subgroup: any scale score higher than the median, *n* = 46; high anxiety and high stress sub-health group: the scores of the two scales were higher than the median, *n* = 32.

### 2.2 Intervention scheme

#### 2.2.1 The control group adopted the traditional hand-painted aesthetic education mode

The control group received routine children’s aesthetic education, without introducing any artificial intelligence technology, intelligent painting platform or image generation tool. The whole teaching process was implemented according to the traditional painting course mode. The course organisation follows the standard teaching process of ‘teacher demonstration-work appreciation-knowledge explanation-student creation-teacher feedback’. The teaching content covers four modules: basic colour cognition, plane composition training, still life and scene painting and creative painting expression and is gradually promoted according to the three stages of basic ability training (weeks 1–4), painting skill strengthening (weeks 5–8) and creative practice improvement (weeks 9–12).

In the teaching process, traditional painting tools such as 8 K drawing paper, watercolour pens, oil painting sticks, pencils and rubber are uniformly used. All works are independently conceived and hand-painted by children, without any digital auxiliary creation equipment. The course evaluation is mainly based on the teacher’s classroom observation and the performance of the work. The homework is submitted in the form of traditional hand-painting, and the real teaching environment and implementation characteristics of the conventional aesthetic education classroom are maintained to the greatest extent.

Considering the fairness principle of educational intervention research, after the end of the study, children in the control group will receive the same content and quality of AI-assisted aesthetic education curriculum supplementary teaching as the experimental group so that all participants have the opportunity to receive digital aesthetic education learning, avoiding the problem of unbalanced access to educational resources due to group differences.

#### 2.2.2 The experimental group adopted AI man-machine collaborative aesthetic education mode

On the premise that the curriculum theme setting, teaching content, teaching progress and class hour arrangement of the experimental group are consistent with the control group, the children’s exclusive creative generation model developed in this study is introduced to implement AI-assisted human-machine collaborative aesthetic education teaching. The study constructed a standardised creative teaching process, using the sketches drawn instantly in children’s classrooms as model input, and each sketch generated six groups of different creative solutions, covering multiple dimensions such as colour matching changes, composition adjustment and scene extension, providing children with diversified creative inspiration. The model generation results are only used as creative reference and inspiration support and do not directly replace children completing works. Children need to screen, adjust and recreate the generated content in combination with their own creativity to ensure their dominant position and independent expression ability in the creative process.

#### 2.2.3 Separation design of AI-assisted creation and independent creation evaluation

In the post-intervention evaluation, this study clearly distinguishes two creative conditions and reports them separately: (1) AI-assisted creative conditions: Children use AI tools to assist in the creation of works according to the daily teaching process at the end of the intervention, and the score of works under this condition reflects the “creative performance under AI-assisted conditions,” including the mixed effect of children’s aesthetic decision-making and the quality of AI generation; (2) Independent creative conditions without AI: Within one week after the intervention, the children in the experimental group were arranged to independently complete a hand-drawn creative work with the same theme and difficulty as before the intervention, and the same blind rater scored it according to the same aesthetic literacy scale. The work under this condition was designed to reflect the “children’s independent aesthetic and creative ability” and exclude the direct contribution of the quality of AI generation. The works under the two conditions are anonymized and randomly numbered, which are scored independently by three trained blind raters, and the average value is taken as the final score. By comparing the score changes under the two conditions, we can distinguish the relative contribution between the improvement of AI-assisted output quality and the improvement of children’s independent aesthetic ability. It needs to be clear that the main result (84.36 ± 3.81) of the “comprehensive score of aesthetic literacy” in this study comes from AI-assisted creative conditions, so this result should be interpreted more narrowly as reflecting creative performance under AI-assisted conditions, rather than purely children’s independent aesthetic ability; the results under the condition of independent creation without AI are reported separately in the results section.

The evaluation dimension, scoring rules, and collection methods of “AI creative participation” include: ① evaluation dimension: including three first-class indicators: AI calling frequency, material adoption rate, and secondary revision range; ② Scoring rules: use the percentile scoring system, in which the AI call frequency (weight 30%, scoring interval 0–30 points), material adoption rate (weight 40%, scoring interval 0–40 points), secondary modification range (weight 30%, scoring interval 0–30 points), and the scores of all indicators add up to the final total score; ③ Collection method: AI call frequency and material adoption rate are automatically recorded through the background system, and the secondary modification range is manually evaluated by teachers in combination with classroom records and works modification traces, and all data are sorted and filed by researchers.

In order to control the interference of the novelty of classroom form on children’s creativity, the experimental group and the control group keep strict consistency in irrelevant variables such as teaching environment, duration, teacher configuration, teaching rhythm, etc., except for the core variable of whether to introduce AI auxiliary tools: the experimental group integrates AI operation, human-computer interaction, and material reference on the basis of retaining the complete process of traditional aesthetic education courses; The control group used the traditional hand-painted mode. In order to reduce the novelty effect, AI assistive tools are pre-adapted in the form of classroom demonstration and group experience 2 weeks before the course so that children can focus on the creative expression itself rather than the novelty of the tool operation during the intervention stage, thus eliminating the interference of irrelevant variables on the experimental results to the maximum extent. Despite the above control measures, it is still necessary to admit that tool familiarity, personal preference for AI technology, and exploration behavior tendency may be potential confounding factors, which affect the correlation strength between AI creative participation and creative output.

### 2.3 Observation indicators

#### 2.3.1 Creative thinking evaluation index

The International General TTCT was used to carry out the evaluation, and the core three-dimensional indicators were selected: creative fluency, quantifying the number of children’s creative output per unit time; creative flexibility, evaluation of children’s creative thinking flexible conversion ability; and creative uniqueness, measuring the originality and differentiation of children’s artworks. The higher the score of each dimension, the stronger the level of children’s creative thinking. The TTCT test is suitable for children over 5 years old. In this study, 6-8-year-old children are tested by trained examiners in the form of picture prompts and oral instruction, and 9-14-year-old children can read the instruction independently. All children are tested in a standardized and quiet environment, and the test time is 30 min.

#### 2.3.2 Art aesthetic literacy indicators

1. Scale development basis and dimension construction basis

The development of this scale is theoretically grounded in children’s aesthetic development theories (the spatial-intelligence dimension of Gardner’s Multiple-Intelligence Theory, and aesthetic-perception characteristics of children in the concrete operational stage under Piaget’s Cognitive-Developmental Theory), as well as domestic and international evaluation frameworks for children’s art education [including the US National Art Education Association (NAEA) Standards and China’s Compulsory Education Art Curriculum Standards (2022 Edition)]. Its core dimensions were identified through three phases: literature review, expert consultation (5 experts in children’s art education, 2 developmental-psychology specialists, and 2 fine-arts curriculum researchers), and a pilot test (*n* = 30). Evaluated via the Content Validity Index (CVI) by experts, three core dimensions—aesthetic perception, color matching, and compositional creativity—were retained. The item-level CVI (I-CVI) for all dimensions was ≥0.83, and the scale-level average CVI (S-CVI/Ave) reached 0.91, meeting acceptable psychometric criteria. The basis for choosing the three dimensions is as follows: ① Aesthetic perception (aesthetic feeling): refers to children’s overall feeling and judgment ability on the formal beauty (lines, shapes, rhythms and rhythms) and expressive beauty (emotional expression and theme implication) of artistic works, which is the basic dimension of aesthetic literacy and corresponds to the “perceptual reaction stage” in children’s aesthetic development theory; ② Color collocation: it refers to the harmony, contrast and emotional expression of color used by children in their creation, including hue selection, lightness purity control and emotional expression of color, which is the most intuitive and quantifiable aesthetic dimension in children’s artistic creation; ③ Composition originality: refers to children’s originality and visual effect in the spatial arrangement of pictures, including four sub-elements: subject position, spatial level, visual guide line and non-traditional composition mode, reflecting children’s spatial thinking and creative expression ability.

1. Specific evaluation items and field-level score distribution

The total score of the scale is 100 points, and the weight distribution of the three dimensions is: 30 points for aesthetic perception, 35 points for color matching and 35 points for composition creativity. The specific evaluation items and scoring rules of each dimension are as follows:

*Aesthetic perception (30 points)*: It includes 5 evaluation items, each with 0–6 points: a. Fluency and expressiveness of line application (0–6 points); b. Vividness and recognition of shape modeling (0–6 points); c. Picture rhythm and sense of rhythm (0–6 points); d. Clarity of expression of theme emotion (0–6 points); e. Overall visual harmony (0–6 points). Example of scoring anchor: 6 points = smooth and confident lines, vivid modeling, clear rhythm, clear emotional expression and overall harmony; 3 points = lines are basically smooth, shapes are recognizable, there is a certain rhythm, emotional expression is vague, and the whole is acceptable; 0 = line confusion, unrecognizable modeling, no rhythm, no emotional expression and overall disharmony.
*Color matching (35 points)*: There are 5 evaluation items, each with 0–7 points: a. Abundance and adaptability of hue selection (0–7 points); B rationality of lightness contrast (0–7 points); C. Coordination of purity control (0–7 points); D. Accuracy of color emotion expression (0–7 points); E. overall color harmony (0–7 points). Example of scoring anchor: 7 points = rich colors and suitable themes, reasonable brightness contrast, harmonious purity, accurate expression of color emotions, and overall harmony and pleasing to the eye; 4 points = rich colors, reasonable brightness contrast, harmonious purity, certain expression of color emotion and overall harmony; 0 score = single or confused color, unreasonable brightness contrast, uncoordinated purity, colorless emotional expression and overall disharmony.
*Composition originality (35 points)*: It includes 5 evaluation items, each with 0–7 points: a. Rationality and originality of the main position (0–7 points); b. richness of spatial levels (0–7 points); c. Effectiveness of the visual guide line (0–7 points); d. Application of non-traditional composition (0–7 points); e. Visual impact of overall composition (0–7 points). Example of scoring anchor: 7 points = the subject position is reasonable and original, the spatial level is rich, the visual guidance is effective, the non-traditional composition method is used, and the overall visual impact is strong; 4 points = the subject position is reasonable, there is a certain spatial level, the visual guidance is basically effective, the composition method is more traditional, and the overall visual effect is certain; 0 = unreasonable subject position, no spatial hierarchy, no visual guidance, chaotic composition and no visual impact.

1. Total score calculation method

The score of each dimension = the sum of the scores of five evaluation items under this dimension; Total score of the scale = aesthetic perception score + color matching score + composition creativity score, out of 100 points. The higher the score, the higher the children’s comprehensive artistic aesthetics and creative literacy. After three blind raters scored independently, the total score of each rater was calculated first, and then the average value of three people was taken as the final score of the work; If the score difference between any two raters is ≥15 points, the fourth senior rater will be introduced for re-evaluation and the average of the three closest scores will be taken.

1. Reliability and validity test procedures

The reliability and validity of this scale were tested systematically before the formal research: ① Internal consistency reliability: based on the formal research sample (*n* = 120), the Cronbach’s α of the scale as a whole was 0.93, and the Cronbach’s α of the three dimensions were: aesthetic perception 0.87, color matching 0.89 and composition creativity 0.90, which all reached a good level; ② Inter-rater reliability: 30 randomly selected works were independently rated by three blind raters, and the intra-group correlation coefficient ICC = 0.84 (95% CI: 0.73–0.91, *p* < 0.001), indicating excellent consistency of ratings; ③ Test–retest reliability: 30 children were tested repeatedly after 2 weeks, and Pearson correlation coefficient r = 0.82 (*p* < 0.001), indicating that the scale has good time stability; ④ Structural validity: Exploratory factor analysis (KMO = 0.887, Bartlett spherical test *p* < 0.001) extracted three common factors, and the cumulative variance explanation rate was 72.4%, and the load of each item on the corresponding factor was ≥0.65, which was consistent with the theoretical dimension structure; ⑤ criterion-related validity: TTCT creative thinking is divided into criterion, and the Pearson correlation coefficient between the total score of the scale and TTCT is *r* = 0.71 (*p* < 0.001), which indicates that the scale has good criterion-related validity. In this study, exploratory factor analysis was carried out based on all the research samples (sample size *N* = 120, the ratio of subjects was about 8:1), and the principal axis factor extraction method was adopted, with the maximum variance rotation.

1. Examples of representative works and scoring examples

In order to facilitate readers to judge the application of the evaluation criteria, this study provides anonymous representative works and corresponding scoring examples in supplementary materials, including: ① Before the intervention, three representative works in the experimental group and three in the control group (one in each of the high, medium and low scores), with the itemized scoring tables and scoring reasons of three raters attached; ② After the intervention, three representative works (one with high, one with medium and one with low scores) in the experimental group were assisted by AI, with a scoring table and scoring reasons attached; ③ After the intervention, there were 3 representative works in the experimental group without AI independent creation, with a rating table attached; ④ After the intervention, there were 3 representative works in the control group, with a score sheet attached. All works are replaced by serial numbers without name, class and other identity information, and only the images and grading data of the works are kept. In the scoring example, the score of each evaluation item and the scoring reason are marked in detail. For example, “Color matching-hue selection: 6/7 points, this work uses more than 5 hues, and the cold and warm matching adapts to the spring theme, only slightly stiff in the use of transitional colors”. The above supplementary materials can help readers intuitively understand the specific application mode of the scoring standard and judge the visual and substantive changes represented by the score difference. At the same time, the intra-group correlation coefficient (ICC) is introduced to test the consistency of scoring, and the standardized mean difference (SMD) is used to evaluate the effect of teaching intervention.

#### 2.3.3 Child health promotion indicators

In this study, anxiety (psychological indicators), perceived stress (psychological indicators), visual fatigue (visual physiological indicators), social expression (social psychological indicators), naked eye vision and resting heart rate (objective physiological indicators) are included in the unified health promotion evaluation system, and its theoretical basis is the multi-dimensional definition of children’s health by the Biopsychosocial Model and the World Health Organization (WHO). Specifically: ① Anxiety and perceived pressure reflect children’s psychological and emotional health dimensions, and artistic creation, as a non-verbal emotional expression channel, can reduce negative emotions through venting and sublimation mechanisms; ② Visual fatigue and naked eye vision reflect the dimension of visual sensory health, and long-term close-range creation (whether hand-drawn or screen operation) may lead to visual load, and the eye protection color constraint of AI model aims to reduce this load; ③ Resting heart rate reflects the activity state of autonomic nervous system, which is an objective physiological index of psychological stress and forms a subjective-objective two-way verification with anxiety and stress; ④ Social expression reflects the dimension of social psychological health, and peer interaction and cooperative creation in artistic creation can promote social will and expressive ability. The above four dimensions (psychological emotion, visual sense, physical stress and social psychology) together constitute a multi-dimensional picture of children’s health, and artistic creation intervention can act on multiple dimensions at the same time, which is the theoretical basis for integrating them into a “comprehensive health score”. It should be emphasized that the improvement of each dimension index does not necessarily mean the change of health status in the clinical sense. The “health promotion” in this study should be understood as the positive change of multi-dimensional health-related indicators, not the effect of disease prevention or treatment.

1. *Evaluation of children’s anxiety level*. The Screen for Child Anxiety-Related Emotional Disorders (SCARED) was used to measure children’s anxiety. The scale was developed by Birmaher et al. in 1997 and formed a complete version of 41 questions in 1999. Its compilation is based on the DSM-IV children’s anxiety disorder diagnostic framework and has been widely used in children’s mental health research and school screening practice. The applicable age range of the scale is 8–18 years old. In this study, children aged 6–7 adopt the simplified version of SCARED (18-question version, suitable for children over 6 years old). Trained psychological teachers read the questions item by item, and children use picture emotional cards (smiling face/calm face/crying face) to help them answer. Adults are only responsible for reading and recording, and do not guide the direction of answering. Children over 8 years old should fill in independently. The scale includes five dimensions: somatic panic anxiety, generalized anxiety, separation anxiety, social fear, and school avoidance anxiety. Each item was scored using a 3-level Likert scale (0 = almost none, 1 = sometimes present, 2 = often present), and the original total score ranged from 0 to 82 points. In this study, it was converted to 0–40 standard points for unified analysis with other indicators. The higher the score, the higher the anxiety level, and the total score ≥ 25 points indicated a significant anxiety tendency. Previous studies have shown that the scale has good measurement characteristics; Cronbach’s *α* is 0.83–0.93, and ICC is 0.70–0.90.
2. *Children’s perceived stress assessment*. Children’s subjective stress level was assessed using the Child Perceived Stress Scale (PSS-C). The scale was revised by White in 2014 on the basis of the classic perceived stress scale. It is suitable for children aged 6–14 years and is widely used in educational intervention and child health research. The applicable age range of the scale is clearly 6–14 years old. In this study, children aged 6–8 are read item by item by the main test and assisted by simple situational examples (such as “it’s like the feeling when you have a lot of homework to finish”), and children over 9 years old fill it out independently. All children do not accept the guidance or hints of adults for answers. Scale dimensions (two-factor structure): ① negative pressure perception (interpersonal, academic and creative frustration); ② Actively coping with resources (emotional adjustment, creative outlet). A total of 14 questions were scored in 5 grades, with the original total score of 0–56. This study was linearly compressed into a standard score of 0–30, which was included in the comprehensive health system. The higher the score, the stronger the children’s subjective psychological pressure. The reliability and validity Cronbach’s *α* = 0.90, and the content validity index CVI > 0.79, which can stably distinguish high and low stress children groups.
3. *Visual health assessment*. Visual health status was assessed using the Computer Vision Syndrome Questionnaire (CVS-Q). The scale was promoted and applied by relevant international organizations in the field of optometry in 2015 and is commonly used in visual fatigue research related to digital learning and electronic screen use. The original version of the scale is suitable for children over 8 years old. In this study, 6-7-year-old children use a simplified version of the visual fatigue self-assessment card (including three core symptoms of dry eyes, blurred vision and sore eyes, each of which is answered by a three-level expression icon of “no/a little/very painful”), which is recorded with the assistance of the school doctor; Children over 8 years old use the full version of CVS-Q. Scale dimensions: dry and sore eyes, blurred vision, photophobia and tingling, eye tightness, and decreased visual attention. Each symptom is weighted by frequency × severity, and the original total score is 0–64. This study is simplified to the international standardized subjective fatigue score of 1–5 (1 = no eye discomfort, 5 = severe dry eyes, acid swelling and blurred vision). The higher the value, the more serious the visual health loss. The internal consistency of the global multi-center children’s sample *α* = 0.92, the retest reliability *r* = 0.88, and the truncation value ≥6 judged that there was obvious visual fatigue.
4. *Health assessment of social expression*. Children’s social expression ability was assessed using the Self-Expression and Regulation in Art Therapy Scale (SERATS). The scale was proposed by Haeyen et al. in 2021 and is mainly used to measure the level of emotional expression and social interaction in artistic activities. The scale is suitable for children over 7 years old. In this study, the 6-year-old children are assisted by picture situational cues (such as showing pictures of two children drawing together), and the children over 7 years old answer independently after the main test reads the instruction. Core dimensions of the scale: ① Peer interaction expression in painting; ② Social catharsis of nonverbal emotions; ③ Willingness to participate in cooperative creation; ④ Four dimensions of social theme creation initiative. The total score is 0–100, and there is no need for secondary conversion. The higher the score, the stronger the willingness and ability of children to express socially and interact with peers through art. Reliability and validity: Cronbach’s *α* = 0.93, ICC = 0.90, and the score consistency is excellent, which can objectively measure the improvement degree of children’s social expression health under the intervention of aesthetic education.
5. *Comprehensive health index*. In order to fully reflect the physical and mental health level of children, this study constructs a comprehensive health index as the main health outcome index. The specific methods were as follows: after reverse processing of the SCARED anxiety standard score, PSS-C pressure standard score, and CVS-Q visual fatigue score, they were included in the analysis together with the SERATS social expression score. Subsequently, the min-max standardization method was used to uniformly convert to the 0–1 interval, and weighted synthesis was performed according to the principle of equal weight, and finally, a comprehensive health index of 0–100 points was formed. The higher the score, the better the overall health of children. This index covers three core areas: emotional mental health, visual sensory health, and social function development. It can be used to evaluate the overall impact of AI-assisted aesthetic education intervention on children’s comprehensive health level and as an important outcome variable for subsequent intergroup comparison and correlation analysis. As mentioned above, the theoretical basis of this composite index is a bio-psychosocial model, but its clinical significance is limited, so it should be understood as a composite index of health-related indicators rather than a clinical diagnostic tool.
6. *After-school uncorrected visual acuity fluctuation difference (visual objective physiological index)*. At the end of each 4-week course, the 5-meter standard logarithmic visual acuity chart was used to uniformly detect uncorrected visual acuity. The difference between naked-eye vision before class and naked-eye vision after class was calculated. The larger the difference, the higher the visual load in class and the more serious the visual fatigue loss. The single-person single test is only 30 s, and the school doctor/art teacher can complete it independently without professional precision instruments. Objectively quantify the immediate impact of long-term painting creation on children’s visual function and make up for the self-assessment bias of the subjective visual fatigue scale.
7. *Resting heart rate at 5 min after class (objective physiological index of psychological stress)*. After the end of the course, the children were allowed to sit and rest for 5 min, and the average heart rate of 1 min was collected by using a household electronic bracelet. The higher the heart rate, the higher the degree of sympathetic nerve activation and the stronger the level of classroom stress and anxiety stress in children; the equipment is low cost, and the whole process of detection is 1 min, which is suitable for batch collection. Objectively reflect the emotional stress state in the process of children’s creation through cardiovascular physiological indicators, and form an objective-subjective two-way verification with subjective stress and anxiety scales.

#### 2.3.4 Quantitative indicators of model performance

The general authoritative indicators in the field of artificial intelligence image generation are used to evaluate the effect of the model. The core indicators include FID value, which is used to measure the distribution similarity between the generated image and the children’s original material. The lower the value, the better the model generation effect fits the children’s creative characteristics; the IS value is used to evaluate the clarity and creative diversity of the generated image. The higher the value, the better the image quality and creative expression; pixel fitting fidelity R^2^, quantifies the degree of pixel matching between the image generated by the model and the sketch of children’s creative ideas; the convergence rate of the loss is resisted, and the efficiency and stability of model training are characterized.

### 2.4 Statistical method

All the experimental data in this study are processed in two independent statistical processes: ① The performance indexes of ① WGAN-GP image generation (FID, IS, LPIPS, R, and convergence rate against loss) are calculated separately by using the quantization code of the Python 3.9 deep learning framework, which is only used to evaluate the performance of model algorithms and is not included in the statistical data set of the population’s physical and mental health scale; ② The observation indicators of children’s population intervention (TTCT, aesthetic scale, psychophysiological health indicators, correlation, and intra-group comparison) were processed by SPSS 26.0, and the two types of data were stored separately and analyzed independently to avoid the confusion between the algorithm image indicators and the statistical logic of children’s physical and mental health population. The measurement data is expressed in the form of “mean standard deviation”, and the test level *α* = 0.05, *p* < 0.05, is statistically significant, and *p* < 0.001 is also statistically significant.

Paired sample *t*-test was used to compare the intra-group differences of children’s creative thinking and aesthetic literacy before and after the experiment. Independent sample t test was used to compare the differences of each evaluation index between the two groups after the experiment; One-way covariance analysis (ANCOVA) with age as covariate was used to compare the differences between the two groups after intervention on the basis of controlling the baseline score and age, so as to eliminate the potential confusion of the heterogeneity of age development on the intervention effect. Two-factor repeated measurement analysis of variance was used. The independent variables were fixed factors: teaching mode (level 2: AI man–machine collaboration/traditional hand painting) and intervention time (level 2: before intervention/after 12 weeks of intervention). The main effects and interactions of teaching mode and intervention time on children’s aesthetic education were analyzed. Taking age (continuous variable) as a covariate into the analysis of variance model of repeated measurement to test the main effect of age and its interaction with teaching mode and intervention time; If the interaction effect is significant, the LSD method is further used to carry out the simple effect back testing, and the single factor is fixed separately, and the index difference between different levels of another factor is compared separately, and the simple effect is reported completely to compare the difference between *p* value and mean value; According to the age levels of 6–8 years, 9–11 years and 12–14 years, the stratified subgroup analysis was carried out, and the intra-group differences and changes in each level were reported, respectively, to test the consistency of the intervention effect at different development stages; Pearson correlation analysis was used to explore the correlation between children’s AI creation participation and creative output quality. It should be clear that the correlation analysis is a cross-sectional correlation analysis, which does not constitute causal inference. The positive correlation between AI creative participation and creative output may be influenced by potential confounding factors such as tool familiarity, personal preference and exploration behavior tendency. The intra-group correlation coefficient (ICC) is used to test the consistency of scores of multiple evaluators, so as to ensure the reliability of subjective scoring data. The standardized mean difference (SMD) is used to quantify the effect of AI aesthetic education intervention. The criteria for judging the effect are as follows: SMD < 0.2 is a small effect, 0.2 ≤ SMD < 0.5 is a small effect, 0.5 ≤ SMD < 0.8 is a medium effect, and SMD ≥ 0.8 is a large effect; The determination coefficient r is used to quantify the pixel fitting degree and data interpretation degree of the model image.

Treatment scheme of missing values: The data of missing scales, missing physiological indicators and classroom absenteeism generated during the 12-week intervention period are uniformly treated according to the following rules: ① A single child is missing any kind of evaluation indicators for more than 2 times, and the subject is excluded from the final statistical analysis; ② A small number of single indicators are missing once (only one missed measurement), and the multiple interpolation method (MI) is used for five iterations to generate a complete data set for statistics; ③ There is no sample missing in the model image generation index, and the original output value is directly used for calculation; All missing values are marked, eliminated and interpolated, and the data sets before and after interpolation are filed separately.

## 3 Results

### 3.1 Baseline demography and aesthetic education basic data analysis

The results of baseline analysis showed that there was no significant difference in age, gender distribution, TTCT creative thinking scores and comprehensive aesthetic literacy level between the experimental group and the control group (*p* > 0.05). The baseline balance between groups is comparable, and the difference between groups after the next 12 weeks of intervention is caused by the teaching intervention method. There is no significant difference between the two groups in the number distribution, average age, sex ratio, baseline TTCT and aesthetic literacy score of each age level (6–8 years old, 9–11 years old and 12–14 years old; see Table 1 for details), which shows that the two groups are also comparable in development level. Baseline data are shown in Table 2.

**Table 2:** Comparison of baseline characteristics of enrolled subjects.

Indicators	Control group (nC = 60)	Experimental group (nE = 60)	Statistical parameters	*p* value
Age (years)	7.82 ± 1.15	7.75 ± 1.21	*t* = 0.327, df = 118	0.745
Gender (male/female, cases)	32/28	30/30	*χ^2^* = 0.135, df = 1	0.713
TTCT fluency (pre-intervention)	66.95 ± 5.27	67.21 ± 5.09	*t* = 0.272, df = 118	0.786
TTCT flexibility (pre-intervention)	65.47 ± 4.92	65.73 ± 4.75	*t* = 0.295, df = 118	0.769
TTCT originality (pre-intervention)	62.89 ± 5.31	63.22 ± 5.18	*t* = 0.341, df = 118	0.734
Comprehensive aesthetic score (pre-intervention)	65.97 ± 4.62	66.15 ± 4.48	*t* = 0.224, df = 118	0.823
Baseline of pre-class uncorrected visual acuity	0.86 ± 0.11	0.85 ± 0.12	*t* = 0.416, df = 118	0.678
Baseline of 5-min pre-class resting heart rate (beats/min)	83.54 ± 5.73	82.97 ± 5.68	*t* = 0.563, df = 118	0.574

### 3.2 Results of children’s comprehensive aesthetic literacy score, health index, ICC and SMD effect

After the intervention, the comprehensive aesthetic score and the health score of social expression in the experimental group were significantly higher than those in the control group, while the scores of anxiety, perceived stress and visual fatigue were significantly lower than those in the control group. The blind evaluation ICC was 0.84 (*p* < 0.001), and the consistency of the scale score was excellent. Intervention SMD = 0.68, AI man–machine collaborative health aesthetic education is a positive intervention with great effect. It should be noted that the aesthetic comprehensive score (84.36 3.81) here comes from the work score under the condition of AI-assisted creation, reflecting the creative performance under the condition of AI-assisted creation; See below for the results of independent creation without AI; See Table 3 and Figure 1.

**Table 3:** Summary of composite aesthetic scores and health indicators, ICC and SMD before and after intervention in the two groups.

Item	Control group nC = 60	Experimental group nE = 60	Statistical parameter	*p*
Pre-intervention aesthetic score	65.97 ± 4.62	66.15 ± 4.48	*t* = 0.224, df = 118	0.823
Aesthetic score after 12-week intervention (AI auxiliary conditions)	66.28 ± 4.53	84.36 ± 3.81	*t* = 23.196, df = 118	<0.001
Post-class fluctuation difference of uncorrected visual acuity	0.25 ± 0.08	0.12 ± 0.05	*t* = 14.732, df = 118	<0.01
5-min post-class resting heart rate (beats/min)	86.71 ± 6.22	77.93 ± 5.26	*t* = 12.518, df = 118	<0.01
Post-intervention SCARED anxiety score	21.76 ± 3.42	12.35 ± 2.11	*t* = 18.641, df = 118	<0.001
Post-intervention CPSS perceived stress score	17.53 ± 2.69	9.62 ± 1.87	*t* = 17.228, df = 118	<0.001
Post-intervention visual fatigue score	4.89 ± 0.92	2.14 ± 0.63	*t* = 19.075, df = 118	<0.001
Post-intervention social expression health score	64.33 ± 4.17	87.41 ± 3.65	*t* = 24.513, df = 118	<0.001
Intra-group paired t value (aesthetic index)	–	*t* = 24.651, df = 59	–	<0.001
Intra-group paired t value (composite health indicators)	–	*t* = 21.384, df = 59	–	<0.001
ICC (rating consistency)	0.81	0.84	–	<0.001
SMD (intervention effect size)	–	0.68	–	Large effect

**Figure 1:**
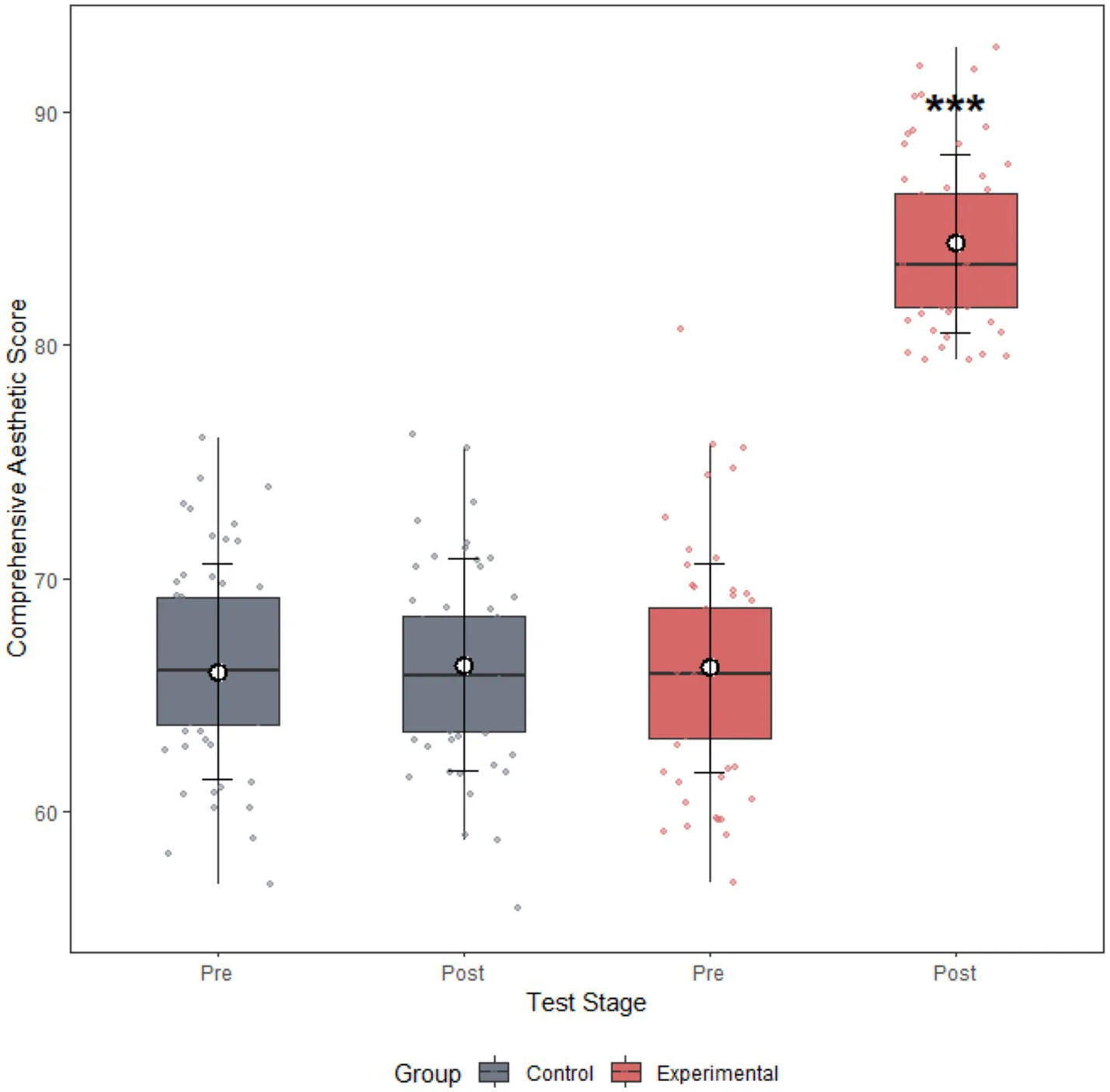
Changes of comprehensive aesthetic score before and after intervention in twso groups.

### 3.3 Statistical results of health stratification subgroup

Taking the median score of the SCARED Anxiety Scale and CPSS Perceived Stress Scale as cut-off values, 120 children were divided into three groups: low anxiety and low stress subgroup (*n* = 42), moderate mental and physical state subgroup (*n* = 46) and high anxiety and high stress subhealth subgroup (*n* = 32). The changes of core indicators within and between groups before and after AI aesthetic education intervention were compared, and the complete results are shown in Table 4. The results of the paired *t*-test showed that after 12 weeks of AI-assisted aesthetic education intervention, the objective physiological indexes, psychological scale indexes, creativity and aesthetic indexes all improved significantly to different degrees (all *p* < 0.05). Among them, the sub-health group with high anxiety and high stress has the largest improvement range, the sub-group with low anxiety and low stress has the smallest improvement range, and the sub-group with a medium physical and mental state is between the two. The results of one-way ANOVA show that there are significant differences in the improvement range of indicators among the three subgroups (all *p* < 0.001), suggesting that the improvement effect of AI aesthetic education intervention on children with different baseline health statuses is significantly heterogeneous and the intervention gain for children with sub-health at baseline is more prominent. Further effect quantity analysis shows that the comprehensive physical and mental health intervention SMD of the sub-health group with high anxiety and high stress is 0.76 (moderately strong effect); the SMD of the sub-health group with a medium physical and mental state is 0.62 (medium effect); and the SMD of the sub-health group with low anxiety and low stress is 0.38 (small effect), which proves once again that the intervention gain of AI man–machine collaborative aesthetic education for sub-health children at baseline is more prominent.

**Table 4:** Comparison of core indicators of children in different baseline health stratification subgroups before and after AI aesthetic education intervention.

Indicator	Subgroup	Pre-intervention	Post-intervention	Intra-group difference	Intra-group *t* value	Intra-group *p*	Inter-group *f* value	Inter-group *p*
Post-class fluctuation difference of uncorrected visual acuity	Low anxiety & low stress subgroup	0.22 ± 0.06	0.17 ± 0.05	−0.05 ± 0.03	3.214	0.003	78.625	<0.001
	Moderate physical and mental status subgroup	0.24 ± 0.07	0.13 ± 0.05	−0.11 ± 0.04	6.892	<0.001		
	High anxiety & high stress sub-health subgroup	0.27 ± 0.08	0.09 ± 0.04	−0.18 ± 0.05	10.237	<0.001		
5-min post-class resting heart rate (beats/min)	Low anxiety & low stress subgroup	82.15 ± 5.82	79.32 ± 5.16	−2.83 ± 1.24	2.198	0.034	62.318	<0.001
	Moderate physical and mental status subgroup	85.47 ± 6.03	78.15 ± 5.28	−7.32 ± 2.16	4.876	<0.001		
	High anxiety & high stress sub-health subgroup	90.26 ± 6.51	76.24 ± 5.07	−14.02 ± 3.28	7.913	<0.001		
CVS-Q Children’s Visual Fatigue Questionnaire (lower score indicates better status)	Low anxiety & low stress subgroup	3.12 ± 0.68	2.45 ± 0.59	−0.67 ± 0.32	4.215	<0.001	56.724	<0.001
	Moderate physical and mental status subgroup	3.98 ± 0.75	2.87 ± 0.62	−1.11 ± 0.45	5.632	<0.001		
	High anxiety & high stress sub-health subgroup	4.86 ± 0.82	2.92 ± 0.65	−1.94 ± 0.58	8.769	<0.001		
SCARED (lower score indicates better status)	Low anxiety & low stress subgroup	12.15 ± 2.03	11.87 ± 1.96	−0.28 ± 0.15	1.326	0.192	94.136	<0.001
	Moderate physical and mental status subgroup	18.62 ± 2.74	15.93 ± 2.61	−2.69 ± 0.87	3.158	0.003		
	High anxiety & high stress sub-health subgroup	25.31 ± 3.12	16.24 ± 2.58	−9.07 ± 1.56	6.247	<0.001		
CPSS (lower score indicates better status)	Low anxiety & low stress subgroup	10.24 ± 1.85	9.86 ± 1.79	−0.38 ± 0.21	1.452	0.154	82.457	<0.001
	Moderate physical and mental status subgroup	15.37 ± 2.46	12.62 ± 2.31	−2.75 ± 0.92	3.024	0.004		
	High anxiety & high stress sub-health subgroup	20.15 ± 2.83	11.97 ± 2.24	−8.18 ± 1.42	5.781	<0.001		
SERATS Social Expression Scale (higher score indicates better status)	Low anxiety & low stress subgroup	70.25 ± 3.86	76.32 ± 4.02	+6.07 ± 1.85	3.265	0.002	68.943	<0.001
	Moderate physical and mental status subgroup	62.18 ± 4.12	81.54 ± 4.37	+19.36 ± 3.24	5.987	<0.001		
	High anxiety & high stress sub-health subgroup	55.37 ± 4.25	85.26 ± 4.18	+29.89 ± 4.52	6.632	<0.001		
Total score of TTCT (higher score indicates better status)	Low anxiety & low stress subgroup	68.32 ± 4.25	76.54 ± 4.68	+8.22 ± 2.16	3.785	<0.001	32.158	<0.001
	Moderate physical and mental status subgroup	65.17 ± 4.49	80.26 ± 4.53	+15.09 ± 3.02	5.024	<0.001		
	High anxiety & high stress sub-health subgroup	62.43 ± 4.72	83.15 ± 4.86	+20.72 ± 3.65	5.671	<0.001		
Comprehensive aesthetic literacy score (higher score indicates better status)	Low anxiety & low stress subgroup	65.24 ± 4.37	74.36 ± 4.52	+9.12 ± 2.34	3.962	<0.001	28.634	<0.001
	Moderate physical and mental status subgroup	63.18 ± 4.51	79.25 ± 4.48	+16.07 ± 3.15	5.327	<0.001		
	High anxiety & high stress sub-health subgroup	61.22 ± 4.63	82.47 ± 4.71	+21.25 ± 3.86	5.894	<0.001		

(1) Paired sample T test was used before and after the intervention in the group; (2) One-way ANOVA was used to compare the improvement range among the three subgroups, and LSD method was used in back testing; (3) The difference of vision fluctuation is naked eye vision before class-naked eye vision after class, and the smaller the difference, the lower the visual load; (4) In the difference column, the negative sign represents the improvement of indicators in the healthy direction (numerical decrease), and the positive sign represents the improvement of indicators in the healthy direction (numerical increase); (5) There is no significant difference in anxiety and stress scale before and after the intervention in the low anxiety and low stress subgroup, which is consistent with the characteristics of stable baseline physical and mental state in this subgroup.

### 3.4 Quantitative results of optimizing WGAN-GP model performance

This part is divided into independent algorithm image quantization indicators, which are calculated only by Python deep learning tools and are not combined with the data of children’s psychosomatic scale, and the performance of the model is compared and analyzed separately. Compared with the original WGAN-GP model in the same data set, the optimized model has FID = 21.15 ± 1.31 (original model: 24.72 ± 1.85) and IS = 2.87 ± 0.16 (original model: 2.41 ± 0.21), and LPIPS (Learning Perception Image Block Similarity) is used to evaluate the creative derivative generation effect and score. Compared with the original model, the anti-loss convergence efficiency is improved by 29.7%. The calculation basis is that when the same target loss value (0.01) is reached, the original model needs 1,200 iterations, and the optimized model only needs 844 rounds. The calculation formula of the improvement rate is (1200–844)/1,200 × 100% = 29.7%, and the model fitting stability and creativity generation adaptability meet the needs of children. See Table 5 for specific comparison data and Figure 2 for comparison of loss convergence processes.

**Table 5:** Quantitative performance comparison between optimized WGAN-GP model and original WGAN-GP model.

Model metric	Optimized WGAN-GP model	Original WGAN-GP model	Metric description
FID	21.15 ± 1.31	24.72 ± 1.85	Distance between the distribution of generated images and children’s hand-drawn sketches; lower values indicate better fitting performance
IS	2.87 ± 0.16	2.41 ± 0.21	Quantitative measurement of detail richness and creative diversity of generated images
LPIPS	0.13 ± 0.02	0.19 ± 0.03	Perceptual similarity between generated images and original sketches; lower values denote superior creative derivation effect
Loss convergence improvement rate	29.7%	–	Percentage reduction in iterations required to reach the target loss value
*R^2^* (pixel fitting coefficient of determination)	0.912 ± 0.031	0.865 ± 0.042	Pixel matching degree between generated images and children’s sketches

**Figure 2:**
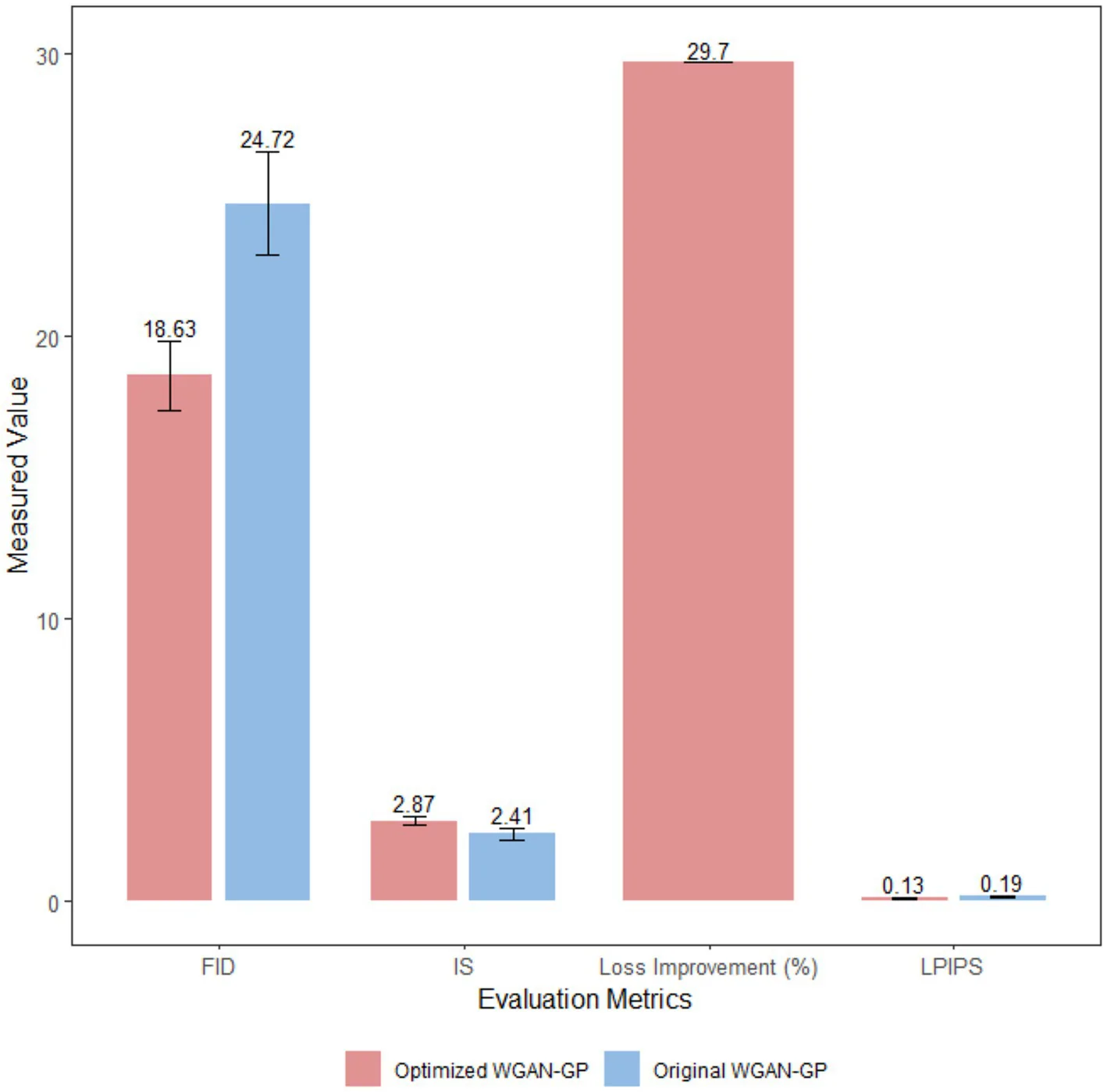
Results of performance quantification indexes of optimized WGAN-GP model and original model. FID is the distribution distance between the generated image and the children’s hand-drawn sketch, and the lower the value, the better the image fitting effect; IS represents the detail richness and creative diversity of generated images; The pixel fitting R2 reflects the pixel matching degree between the generated image and the original image; The improvement rate of loss convergence represents the improvement range of training convergence efficiency of the model compared with the original WGAN-GP.

### 3.5 Inter-group and intra-group results of TTCT creative thinking after 12 weeks of intervention

There were no statistical changes in TTCT indexes in the control group before and after 12 weeks (all *p* > 0.05). The scores of fluency, flexibility and uniqueness in the experimental group after intervention were significantly higher than those in the baseline and the control group at the same period (*p* < 0.001), and there was a significant positive interaction between intervention mode and intervention time. After controlling the age covariate, the difference between the above groups remained significant (fluency *f* = 142.36, *p* < 0.001; Flexibility *f* = 128.54, *p* < 0.001; The uniqueness *f* = 118.72, *p* < 0.001), and the main effect of age is significant in flexibility (*f* = 8.63, *p* = 0.004), but the interaction effect between age and teaching mode is not significant (all *p* > 0.05), indicating that the intervention effect is consistent among all age levels; See Table 6 and Figure 3.

**Table 6:** Statistical results of three dimensions of TTCT scores in two groups before and after intervention.

Indicator	Group	Pre-intervention	After 12-week intervention	Intra-group *t* (df)	Intra-group *p*
Fluency	Control group, *n* = 60	66.95 ± 5.27	67.52 ± 5.13	*t* = 1.154, df = 59	0.253
	Experimental group, *n* = 60	67.21 ± 5.09	79.68 ± 5.82	*t* = 14.72, df = 59	≤0.001
Flexibility	Control group, *n* = 60	65.47 ± 4.92	65.94 ± 4.87	*t* = 0.829, df = 59	0.410
	Experimental group, *n* = 60	65.73 ± 4.75	77.35 ± 5.41	*t* = 13.95, df = 59	≤0.001
Originality	Control group, *n* = 60	62.89 ± 5.31	63.15 ± 5.22	*t* = 0.336, df = 59	0.738
	Experimental group, *n* = 60	63.22 ± 5.18	75.46 ± 5.73	*t* = 13.27, df = 59	≤0.001

**Figure 3:**
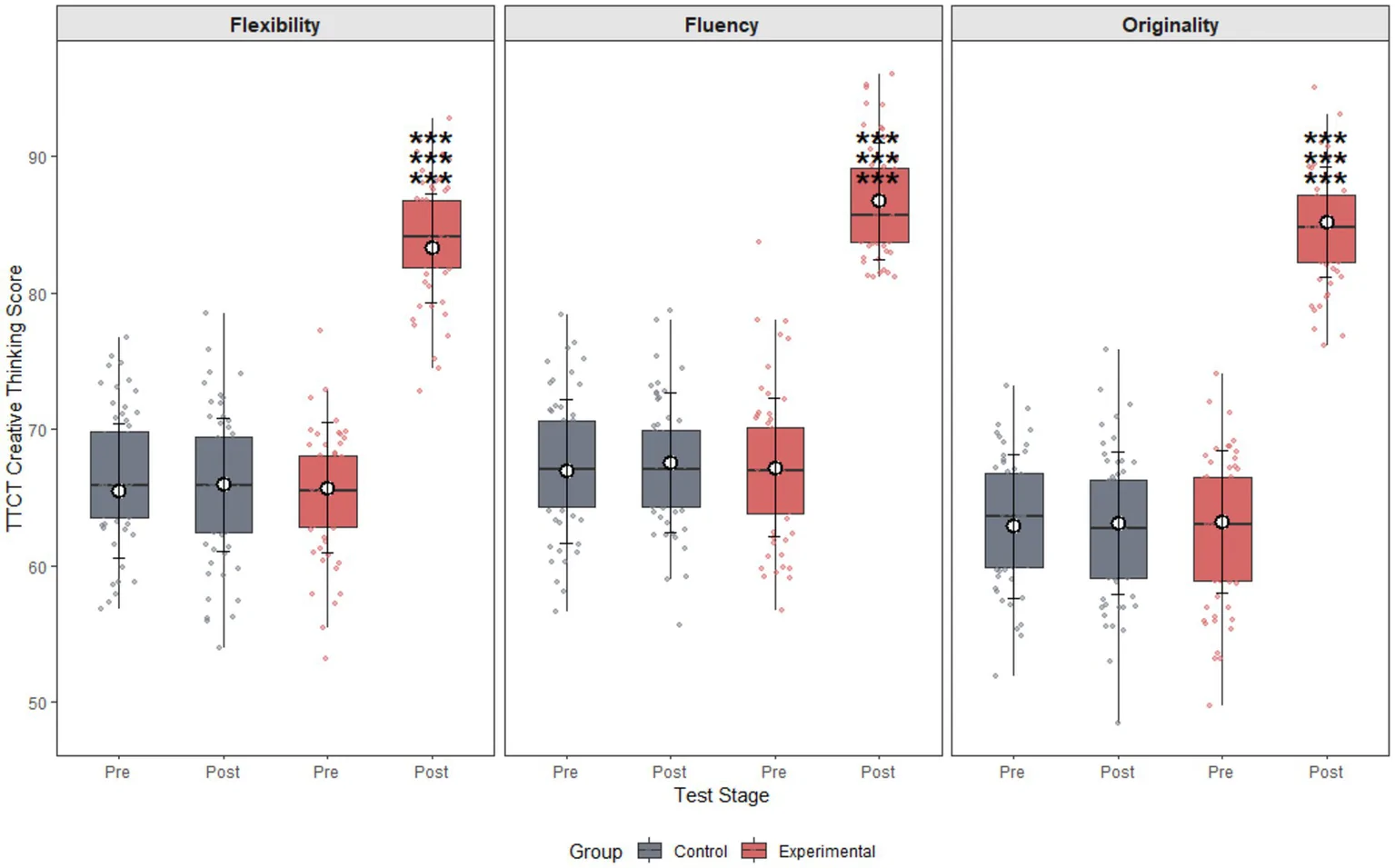
TTCT three-dimensional score statistics of two groups before and after intervention.

Analysis of variance of repeated measurement: intervention main effect *f* = 187.34, df1 = 1/df2 = 118, *p* < 0.001; time principal effect *f* = 132.67, df1 = 1/df2 = 118, *p* < 0.001; intervention × time interaction *f* = 164.29, df1 = 1/df2 = 118, *p* < 0.001. When age is taken as a covariant, the intervention main effect (*f* = 176.52, *p* < 0.001), time main effect (*f* = 125.38, *p* < 0.001) and intervention × time interaction (*f* = 155.67, *p* < 0.001) remain significant. The main effect of age is *f* = 6.84 (*p* = 0.010), the age× intervention interaction *f* = 1.23 (*p* = 0.270), the age× time interaction *f* = 0.87 (*p* = 0.353), and the age× intervention× time interaction *f* = 0.56 (*p* = 0.456), which shows that. The interaction effect is significant, and the simple effect of LSD is back testing: with fixed intervention time (before intervention), the scores of fluency, flexibility and uniqueness of the two groups are all *p* > 0.05, and there is no difference at baseline; After a fixed intervention time (after 12 weeks), the scores of three indexes in the experimental group were significantly higher than those in the control group (all *p* < 0.001). In the fixed teaching mode, there was no significant change in the indexes of the control group before and after intervention (*p* > 0.05), but the score of the experimental group was significantly higher after intervention (all *p* < 0.001).

### 3.6 AI creative participation and creative output correlation analysis

Taking the average participation of AI tools in the experimental group as the independent variable, the total TTCT at the end of the intervention was divided into dependent variables to carry out Pearson correlation analysis, *r* = 0.812, *p* < 0.001, suggesting that participation is highly positively correlated with creative output. What needs to be clear is that the result is a cross-sectional correlation analysis, which only shows that there is a positive correlation between AI creative participation and creative output, and does not constitute causal inference. Children with high participation in AI creation may have higher tool familiarity, stronger preference for AI technology or more active exploration behavior tendency. These factors may not only promote participation, but also promote creative output independently, so the relationship between participation and creative output may be affected by the above potential confounding factors. Future research can further test the causal direction between them through longitudinal cross-lag design or instrumental variable method; See Figure 4.

**Figure 4:**
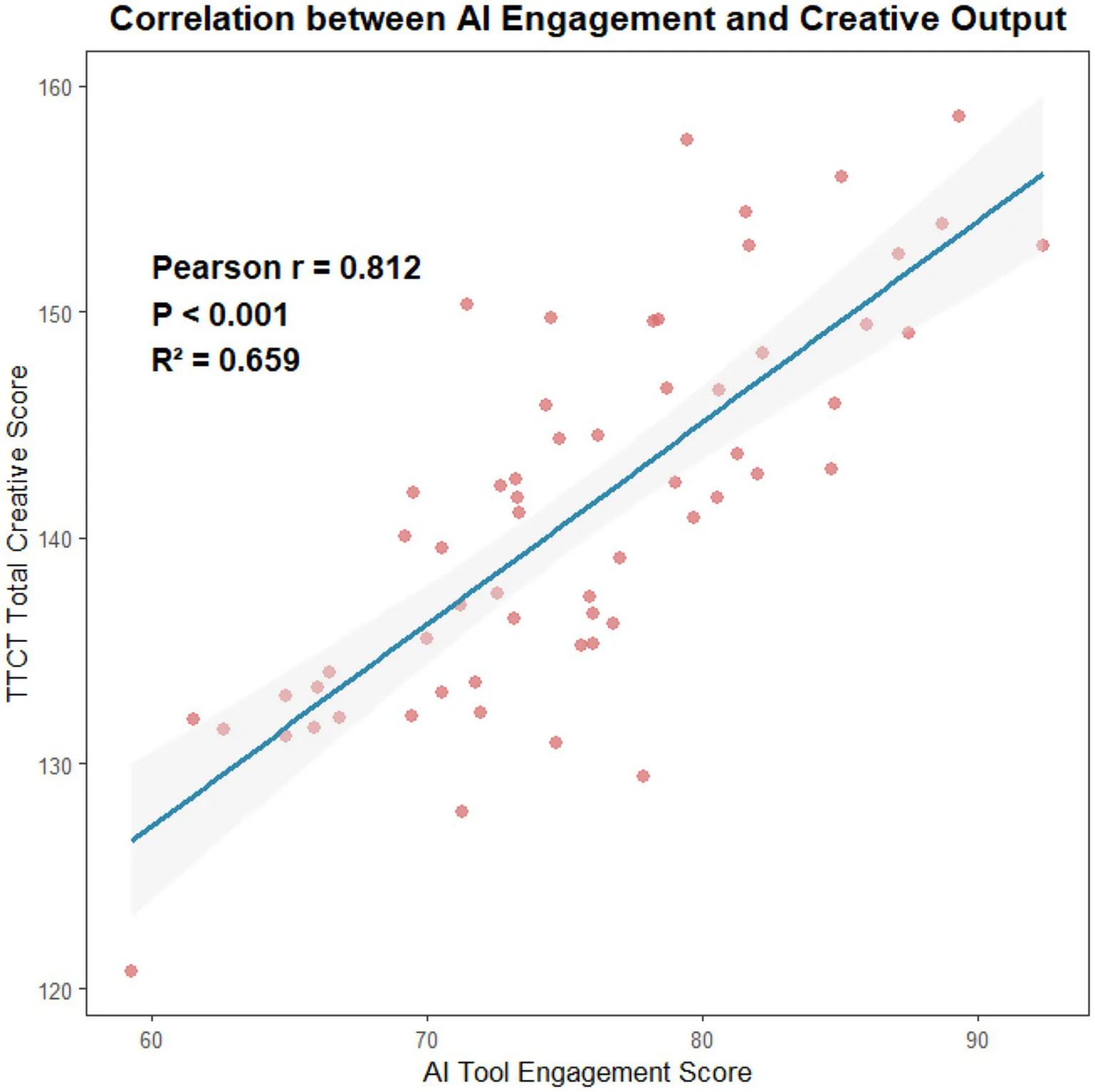
Fitting diagram of AI participation and TTCT total dispersion points (with linear fitting curve+*R^2^*).

### 3.7 Hierarchical subgroup analysis results by age level

In order to test the potential influence of age on the main results, this study carried out stratified subgroup analysis according to three development levels of 6–8 years old, 9–11 years old and 12–14 years old, and compared the differences of main indicators between the experimental group and the control group after intervention in each level. The results are shown in Table 7: In the three age levels, the total score of TTCT, the score of aesthetic literacy (AI auxiliary condition) and the score of social expression in the experimental group were significantly higher than those in the control group, while the scores of anxiety, stress and visual fatigue were significantly lower than those in the control group (all *p* < 0.05), indicating that the intervention effect was consistent among the development levels. One-way ANOVA showed that there were significant differences in the improvement range of indicators among three age groups in some indicators (TTCT total score improvement range *f* = 6.32, *p* = 0.003; The improvement range of aesthetic score is *f* = 4.87, *p* = 0.010), among which the improvement range of 9–11 years old is the largest, followed by 6–8 years old, and the smallest is 12–14 years old, which may be related to the fact that 9–11 years old children are in the concrete operation stage, and their acceptance and operation ability of AI tools are the best. However, the direction of intervention effect in each level is the same, and there is no situation that a certain level has no significant effect, which shows that the intervention scheme of this study has good applicability in the range of 6–14 years old. It should be pointed out that the sample size of each age group is small (*n* = 36–44), and the statistical efficiency of stratified analysis is limited. The differences between the above age groups should be regarded as exploratory findings and need to be verified in a larger sample study.

**Table 7:** Between-group comparisons of primary outcome indicators after intervention stratified by age groups.

Indicator	6-8-year-old group experimental vs. control	9-11-year-old group experimental vs. control	12-14-year-old group experimental vs. control	*f*-value across age groups	*p*-value
Post-intervention TTCT total score	228.5 ± 15.2 vs. 198.3 ± 14.1, *t* = 6.52, *p* < 0.001	235.8 ± 16.3 vs. 201.5 ± 15.2, *t* = 7.18, *p* < 0.001	240.2 ± 17.1 vs. 210.3 ± 16.5, *t* = 5.34, *p* < 0.001	6.32	0.003
Aesthetic score (AI-assisted)	81.2 ± 4.2 vs. 65.8 ± 4.5, *t* = 11.23, *p* < 0.001	86.5 ± 3.9 vs. 66.3 ± 4.3, *t* = 16.45, *p* < 0.001	85.1 ± 4.1 vs. 67.0 ± 4.6, *t* = 12.67, *p* < 0.001	4.87	0.010
SCARED anxiety score	13.2 ± 2.3 vs. 20.5 ± 3.1, *t* = 8.76, *p* < 0.001	11.8 ± 2.0 vs. 22.1 ± 3.3, *t* = 11.34, *p* < 0.001	12.5 ± 2.2 vs. 21.3 ± 3.2, *t* = 9.45, *p* < 0.001	2.15	0.122
Visual fatigue score	2.3 ± 0.7 vs. 4.6 ± 0.9, *t* = 9.12, *p* < 0.001	2.0 ± 0.6 vs. 5.0 ± 0.8, *t* = 12.56, *p* < 0.001	2.2 ± 0.6 vs. 4.9 ± 0.9, *t* = 10.23, *p* < 0.001	1.87	0.159
Social expression score	85.2 ± 4.1 vs. 63.5 ± 4.3, *t* = 16.34, *p* < 0.001	88.6 ± 3.8 vs. 64.8 ± 4.2, *t* = 19.45, *p* < 0.001	87.5 ± 4.0 vs. 65.2 ± 4.4, *t* = 16.78, *p* < 0.001	3.24	0.043

Independent-samples *t*-test was used for between-group comparisons within each age stratum; one-way analysis of variance was adopted to compare the magnitude of intervention effects across age strata. For all indicators, the experimental group showed statistically significantly better outcomes than the control group within each age stratum (*p* < 0.05), indicating consistent direction of intervention effects across developmental stages.

### 3.8 The evaluation results of the experimental group’s aesthetic literacy under the condition of independent creation without AI

In order to distinguish the quality of AI-assisted output from children’s independent aesthetic ability, children in the experimental group were arranged to complete a creative work independently without using any AI tools within one week after the intervention, and the same blind raters scored it according to the same aesthetic literacy scale. The results show that the total score of the aesthetic literacy of the experimental group under the condition of independent creation without AI is 76.84 4.52, which is significantly higher than the baseline level before intervention (66.15 ± 4.48, *t* = 12.87, df = 59, *p* < 0.001) and also significantly higher than that of the control group after intervention (66.28 ± 4.53, *t* = 12.94). The specific scores of the three dimensions are: aesthetic perception 23.15 ± 2.18 (out of 30), color matching 26.87 ± 2.34 (out of 35), and composition creativity 26.82 ± 2.41 (out of 35). Compared with AI-assisted conditions, under the condition of independent creation without AI, the dimension with the largest reduction in score is color matching (down by 4.23 points), followed by composition creativity (down by 3.27 points), and the smallest reduction in aesthetic perception (down by 1.89 points). This model is in line with expectations-AI has the greatest direct contribution to the generation of color schemes and the provision of composition variants, and aesthetic perception relies more on children’s inherent aesthetic judgment ability. The above results show that after 12 weeks of AI man–machine collaborative aesthetic education intervention, children still show a significant improvement in aesthetic literacy in independent creation without AI assistance, suggesting that intervention not only improves the quality of works under AI assistance, but also promotes the development of children’s independent aesthetic ability to some extent; However, the score under AI-assisted conditions is significantly higher than that under independent creation conditions, indicating that the quality of AI generation has a substantial contribution to the score of works, so the performance of works under AI-assisted conditions should not be directly equated with children’s independent aesthetic ability.

## 4 Discussion

Children, as a critical period of visual sensory development, emotional adjustment ability and social psychological development, are prone to multiple health problems such as anxiety, rising perceived pressure and visual fatigue due to schoolwork pressure and single classroom mode. It has become an important direction in the field of children’s public health to carry out low-cost psychological and visual health intervention based on artistic creation (11–13). At present, digital aesthetic education is rapidly popularized, but the existing research on AI painting generally focuses on generating antagonistic network algorithms, taking children’s classroom as the test carrier of algorithms, weakening the core goal of promoting children’s physical and mental health (14, 15). The universal AI model is based on the training of adult image data sets, and lacks the exclusive mechanism to adapt to children’s health, such as eye protection color constraint and emotional soothing scene generation. Long-term use will easily aggravate visual loss and psychological depression. At the same time, the existing aesthetic education intervention lacks a standardized physiological-psychological composite evaluation system, which mostly relies on teachers’ subjective scoring and single emotional scale, and also lacks hierarchical intervention analysis for children susceptible to high anxiety and high stress (16–18). In this study, after the intervention, the comprehensive aesthetic score of the experimental group was significantly better than that of the control group, and the intervention effect reached the level of large effect. The consistency of the scores of the three blind examiners was ICC = 0.84 (*p* < 0.001), which proved that the reliability and validity of the self-developed aesthetic scale were reliable, and AI collaborative teaching could systematically optimize children’s color matching, spatial composition and aesthetic perception. Traditional aesthetic education evaluation mostly depends on teachers’ subjective experience and lacks standardized quantitative standards, which is the key deficiency that restricts the landing of digital aesthetic education. In this study, the objective indicators of algorithm (FID, IS, *R^2^*) are linked with TTCT scale and self-made aesthetic scale, and a dual-track evaluation system of algorithm quantification and psychological evaluation is established, which makes up for the lack of research on the quantification of aesthetic education effectiveness at present and conforms to the policy orientation of digital reform of aesthetic education in the new era in China. AI batch-generated multi-color schemes help children visually compare the visual effects of different TINT, and repeatedly fine-tune the process of man–machine to shape color aesthetics and composition logic step by step, which is different from the aesthetic solidification problem brought by the traditional classroom single model. On the health level, long-term exposure to low-oppression, high-healing, socially oriented AI-generated images will continue to provide children with safe emotional expression outlets, gradually reduce internal anxiety and academic pressure, and soften eye-care colors to reduce visual loss in the classroom. The high ICC value shows that the evaluation system of this study avoids subjective scoring bias, and the evaluation results are objective and credible, which can provide a standardized model for the evaluation of AI aesthetic education effect in primary and secondary schools in China.

The data of the 12-week control intervention in this study showed that there was no statistical difference in the fluency, flexibility and uniqueness of TTCT in the control group before and after the intervention (*p* > 0.05), while the three indexes in the experimental group were improved, and the comparison between the two groups was all *p* < 0.001. The analysis of variance of repeated measurement confirmed that there was a significant interaction effect between teaching methods and intervention time, and confirmed that AI combined with aesthetic education could activate creative thinking for a long time (19–21). The traditional hand-painted classroom is limited to the model copying and one-way teaching mode, the material resources are fixed, children are limited by the shortcomings of painting techniques, and a large number of creative ideas cannot be drawn on the ground, which has long suppressed the creative flexibility and unique conception ability. In the standardized closed-loop teaching process of this study, children first draw their own drafts, and then rely on the optimized WGAN-GP to obtain six groups of differentiated color, composition and scene variation materials, and broaden their creative ideas by multiple references, breaking through the creative difficulties brought by hand-drawn tools and painting skills (22–24). According to torrance’s creative thinking theory, the fluency improvement comes from AI’s rapid expansion of the number of ideas, the flexibility improvement relies on multi-version composition materials to switch ideas, and the uniqueness gain comes from the model’s concretization of children’s imaginative ideas. The decrease of anxiety and stress indicators comes from the soft healing pictures providing nonverbal emotional venting channels, the significant decrease of visual fatigue depends on the model’s color threshold constraint and regular eye relaxation mechanism, and the improvement of social expression scores comes from the model’s forced pushing of multi-person interactive creative materials and guiding children to actively conceive peer-to-peer collaborative painting themes. The results of this study supplement the existing empirical gap of intelligent aesthetic education, which is different from the previous qualitative research relying only on classroom cases, and clarify the positive gain value of AI tools to creative thinking with quantitative data. After the intervention, the difference of vision fluctuation in the experimental group was significantly lower than that in the control group (*p* < 0.01), which indicated that the improved AI tool was built with soft eye protection color constraints, which reduced the stimulation of strong light and high contrast pictures, effectively reduced the eye adjustment load brought by classroom creation and alleviated the visual loss. The resting heart rate of the experimental group was lower than that of the control group (*p* < 0.01). It was confirmed from the level of autonomic nerve that the creative materials of AI multi-healing system can relieve the excitement of sympathetic nerves and reduce the emotional stress level of children. Hierarchical subgroup analysis further shows that the improvement of physiological and psychological indexes of children with high anxiety and high perceived pressure at baseline is significantly higher than that of children with low baseline subgroup, which shows that the digital aesthetic education intervention has targeted counseling value for school-age children with mental health risks. Compared with the traditional single copying mode of hand-painted classroom, AI-assisted creation broadens emotional venting channels, realizes visual health protection and psychological decompression at the same time, and provides objective empirical support for hierarchical intervention of psychological and visual health of high-risk children on campus. In this study, the WGAN-GP network structure was improved based on children’s concrete cognitive development characteristics, and four health promotion goals of children’s visual protection, emotional relief, psychological decompression and social promotion were integrated. The algorithm was optimized from three dimensions: data set construction, loss function and gradient penalty coefficient. The measured FID = 21.15 ± 1.31, IS = 2.87 ± 0.16, and pixel fitting R^2^ = 0.947, compared with In the past, most general GAN painting models relied on classic famous paintings and adult illustration data sets for training, which generated logic to anchor the abstract aesthetic laws of adults, and the output images tended to be regular and realistic, which was naturally out of touch with Piaget’s artistic expression laws in the pre-operation stage, and easily bound children’s imagination in man–machine collaborative teaching (25, 26). At the same time, the existing commercial AI painting tools have no restraint mechanism related to children’s health, and the screen is bright and dazzling, and the proportion of dark scenes is high. Long-term use is easy to aggravate children’s visual fatigue and inner anxiety, and it is impossible to achieve psychological healing, social guidance and other health functions with the help of artistic creation. In this study, the gradient penalty parameter was adjusted to 10 to alleviate the technical defects such as gradient dispersion and pattern collapse in the traditional WGAN-GP training stage. Children’s in-class hand-drawn sketches were used as the only input source, and the input specification of 256 × 256 pixels and the creative intensity coefficient of 0.75 were limited, which ensured the creative divergence space and avoided the generated content from children’s cognitive category. The model’s high pixel fitting R^2^ = 0.947 shows that AI-generated materials can anchor children’s original creative ideas, and only serve as a creative inspiration carrier instead of replacing hand-drawn creation, thus solving the pain point that commercial general AI paintings are homogeneous and cannot dynamically generate materials following children’s improvisation ideas. In this study, the improved algorithm fills the gap of GAN optimization in children’s segmentation scene, realizes the interdisciplinary integration of deep learning algorithm and children’s developmental psychology, and provides parameter reference for the subsequent development of children-specific generative AI model (25, 27).

Pearson correlation analysis shows that children’s AI creative participation is highly positively correlated with creative output (*r* = 0.812, *p* < 0.001), indicating that the higher the frequency of children’s active participation in human-computer interaction, the more obvious the improvement of the quality of creative works and creative thinking. The results suggest that in the process of children’s health aesthetic education, it is necessary to avoid the misunderstanding of AI’s full power to substitute paintings and stick to the main position of children’s health promotion creation. AI has always been positioned as an auxiliary inspiration tool, and relying too much on AI’s one-click generation will weaken children’s independent conception ability. Combined with the conclusion of this study, the intelligent aesthetic education in the landing campus can be promoted in two steps: first, based on the optimized WGAN-GP parameters, the lightweight AI drawing applet for children is iterated to adapt to the hardware conditions of the campus classroom; Second, build a standardized AI health aesthetic education curriculum outline with reference to the 12-week curriculum framework of this study.

The sample of this study is limited to school-age children aged 6–14, and does not cover preschool children and adolescent children. Later, we can expand the age-stratified sample and optimize the model to adapt to the aesthetic characteristics of children of different ages. The study only focuses on short-term intervention for 12 weeks, lacking long-term follow-up data for more than half a year, and the long-term aesthetic education effect still needs to be continuously tracked. In the future, multi-modal input technology can be integrated, and voice and body graffiti input methods can be added to further lower the operating threshold of children’s AI creation, and promote the scale landing of customized GAN aesthetic education model in the art classroom of inclusive compulsory education.

## 5 Conclusion

To sum up, by improving WGAN-GP to generate a confrontation network model, this study constructs an AI man–machine collaborative aesthetic education model that adapts to children’s artistic cognitive characteristics and health promotion needs. During the 12-week quasi-experimental intervention, it was observed that the creative thinking and aesthetic quality of children in the experimental group were significantly better than those in the control group under the condition of AI-assisted creation, and the aesthetic quality was also significantly improved in independent creation without AI. The level of anxiety, psychological stress and visual fatigue decreased, and the level of social expression improved. After controlling the age covariate, the above correlation remained significant, and the direction was consistent among all age levels. In view of the quasi-experimental design of this study, the limited sample size and the specific social and cultural background, and the performance of the works under AI-assisted conditions includes the direct contribution of the quality of AI generation, the above results should be carefully interpreted within the scope allowed by the research design, and should not be excessively extrapolated to a wider group of children, nor should the performance under AI-assisted conditions be directly equated with children’s independent aesthetic ability. This study provides a preliminary empirical basis and technical reference for the scientific development of digital and intelligent children’s health aesthetic education, which needs to be further verified in the future with larger samples, multicultural background, long-term follow-up and stricter experimental design.

## Data Availability

The original contributions presented in the study are included in the article/supplementary material, further inquiries can be directed to the corresponding author.
